# p53-inducible SESTRINs might play opposite roles in the regulation of early and late stages of lung carcinogenesis

**DOI:** 10.18632/oncotarget.27367

**Published:** 2019-12-10

**Authors:** Boxiao Ding, Alexander Haidurov, Ayesha Chawla, Anita Parmigiani, Gerarda van de Kamp, Alexandra Dalina, Fang Yuan, Jun Hee Lee, Peter M. Chumakov, Steven R. Grossman, Andrei V. Budanov

**Affiliations:** ^1^Engelhardt Institute of Molecular Biology, Center for Precision Genome Editing and Genetic Technologies for Biomedicine, Moscow, Russia; ^2^School of Biochemistry and Immunology, Trinity Biomedical Sciences Institute, Trinity College Dublin, Dublin 2, Ireland; ^3^Department of Human and Molecular Genetics, Virginia Commonwealth University, Richmond, VA, USA; ^4^Department of Internal Medicine, Virginia Commonwealth University, Richmond, VA, USA; ^5^Wright Center for Clinical and Translational Research, Virginia Commonwealth University, Richmond, VA, USA; ^6^VCU Massey Cancer Center, Virginia Commonwealth University, Richmond, VA, USA; ^7^Department of Molecular and Integrative Physiology, University of Michigan, Ann Arbor, MI, USA; ^*^These authors contributed equally to this work

**Keywords:** SESTRINs, lung cancer, mouse model, mTORC1/2, tumor suppression

## Abstract

SESTRINs (SESN1-3) are proteins encoded by an evolutionarily conserved gene family that plays an important role in the regulation of cell viability and metabolism in response to stress. Many of the effects of SESTRINs are mediated by negative and positive regulation of mechanistic target of rapamycin kinase complexes 1 and 2 (mTORC1 and mTORC2), respectively, that are often deregulated in human cancers where they support cell growth, proliferation, and cell viability. Besides their effects on regulation of mTORC1/2, SESTRINs also control the accumulation of reactive oxygen species, cell death, and mitophagy. SESN1 and SESN2 are transcriptional targets of tumor suppressor protein p53 and may mediate tumor suppressor activities of p53. Therefore, we conducted studies based on a mouse lung cancer model and human lung adenocarcinoma A549 cells to evaluate the potential impact of SESN1 and SESN2 on lung carcinogenesis. While we observed that expression of SESN1 and SESN2 is often decreased in human tumors, inactivation of Sesn2 in mice positively regulates tumor growth through a mechanism associated with activation of AKT, while knockout of Sesn1 has no additional impact on carcinogenesis in Sesn2-deficient mice. However, inactivation of SESN1 and/or SESN2 in A549 cells accelerates cell proliferation and imparts resistance to cell death in response to glucose starvation. We propose that despite their contribution to early tumor growth, SESTRINs might suppress late stages of carcinogenesis through inhibition of cell proliferation or activation of cell death in conditions of nutrient deficiency.

## INTRODUCTION

Lung cancer is the major cause of cancer-related mortality in the US and other Western countries with a 5-year survival rate of less than 18% [1]. Morphologically, lung cancers are subdivided into two major groups – small cell lung cancer and non-small cell lung cancer (NSCLC), with the latter responsible for ~85% of lung cancer cases [2]. Tumor suppressor protein p53 is the key tumor suppressor in the lung that is found mutated in more than 50% of human lung cancers [3]. As shown in lung cancer models in mice, inactivation of p53 strongly accelerates tumor growth and progression and notably shortens the lifespan of the cancer-bearing mice [4]. The major activity of p53 responsible for its tumor suppressor function is transcriptional regulation of thousands of genes among which at least a hundred are bona fide direct p53 targets [5]. Therefore, through activation of its targets p53 is capable of inhibiting cell proliferation, inducing cell death, and suppressing the production of reactive oxygen species [6, 7]. However, the precise mechanism of suppression of carcinogenesis by p53 is still unknown as inactivation of distinct p53 target genes such as those responsible for the induction of cell death, cell cycle arrest, and senescence does not recapitulate the cancer-prone phenotype of p53-deficient mice [8]. Therefore, the characterization of p53 targets in appropriate transgenic cancer models is a valuable tool to understand the mechanisms responsible for tumor-suppressive functions of p53. An NSCLC model in mice based on lung-specific activation of the mutant Kras^G12D^ oncogene is widely used to understand the critical steps of the development of lung cancer by determining the impact of various tumor suppressors and oncogenes [9, 10], however, the role of p53 in suppression of lung cancer is still far from being fully understood.

We have previously characterized a family of genes encoding evolutionarily conserved proteins called SESTRINs [11, 12]. Mammalian genomes contain three genes encoding highly-homologous SESN1-3 proteins [13]. The expression of mammalian SESTRINs is activated by different stress insults such as hypoxia/ischemia, DNA-damage, oxidative stress, and metabolic derangements [12, 13]. Interestingly, p53 activates transcription of two out of three members of the SESTRIN family, SESN1 and SESN2, in response to genotoxic and oxidative stress insults [11, 14, 15], however, the activation of SESN2 in response to metabolic derangements is p53-independent [16].

SESTRINs regulate cellular homeostasis through control of mechanistic target of rapamycin (mTOR) kinase complexes 1 and 2 (mTORC1 and mTORC2) [14, 15]. The mTORC1 and mTORC2 kinases have distinct substrate specificities and regulate different, although interwoven, processes in the cell [17]. In the conditions when cells are supplied with an excess of nutrients in the presence of growth factors mTORC1 supports anabolism and cell growth and suppresses autophagy. Its major effects are mediated by phosphorylation of proteins involved in the regulation of protein synthesis such as 4EBP1 and p70S6K, with subsequent phosphorylation of ribosomal S6 protein by p70S6K, and the components of ULK1 kinase that play a critical role in the regulation of autophagy [17]. mTORC2, in turn, regulates cellular processes through phosphorylation of the members of the AGC kinase family (AKT, PKC, and SGK) and AKT plays a prominent role in the regulation of glucose metabolism, cell growth, and viability [18].

SESTRINs play opposing roles in the regulation of both mTOR-containing complexes: suppressing mTORC1 [15, 19–21] but activating mTORC2 [12, 22, 23]. SESTRINs inhibit mTORC1 through activation of AMPK kinase and inhibition of the GATOR2 protein complex, the inhibitor of the GATOR1 protein complex. GATOR1 works as a GTPase activating protein for RagA/B that forms heterodimers with RagC/D and the RagA/B:C/D complexes regulate the translocation of mTORC1 to lysosomes, where mTORC1 is activated by the small GTPase Rheb [24, 25]. The mechanism of mTORC2 activation by SESTRINs can be mediated by suppression of the mTORC1-activated negative feedback loop [26], though several recent reports demonstrated mTORC1-independent effects of SESTRINs on mTORC2 activation [22, 27]. Both mTORC1 and mTORC2 complexes are often found activated in human tumors supporting tumor growth and proliferation and affecting tumors’ susceptibility to anticancer treatment [28]. Moreover, SESTRINs may modulate lung carcinogenesis through the regulation of cell death in an mTOR-independent manner [16, 29]. In this work we have evaluated the impact of the p53-regulated SESN1 and SESN2 proteins on the regulation of tumor growth in a mouse model of lung cancer and determined the role of SESTRINs in the control of proliferation and cell death of established human lung adenocarcinoma A549 cells. Although inactivation of Sesn2 in mice led to inhibition of tumor growth, inactivation of either SESN1 or SESN2 in A549 cells stimulated cell proliferation and protected cells against cell death induced by glucose starvation.

## RESULTS

### Expression of SESN1 and SESN2 is decreased in human lung cancers

**Figure 1:**
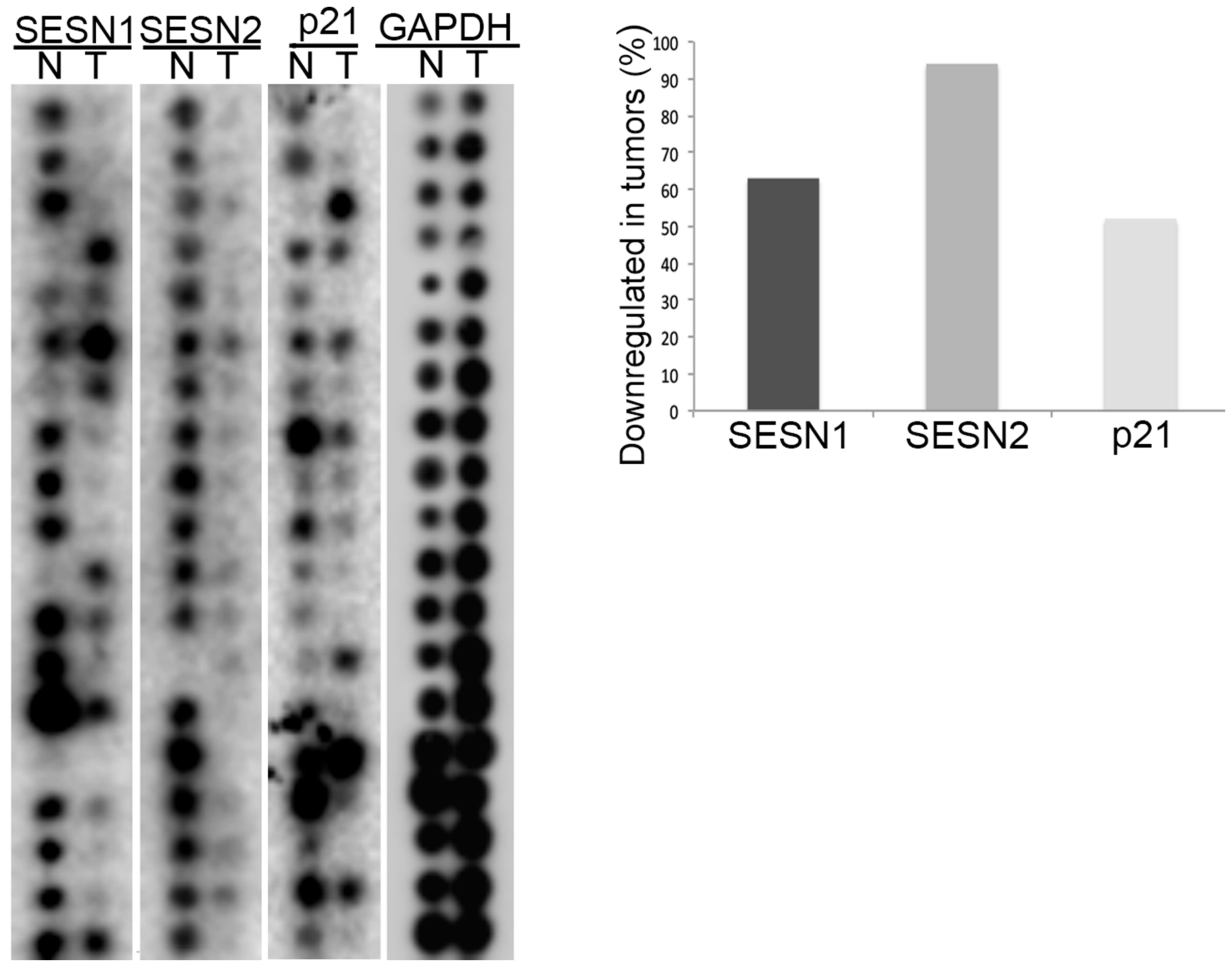
The expression of SESN1 and SESN2 genes is decreased in human lung tumors. The Cancer Profiling Expression Array (Clontech) was hybridized with ^32^P-labelled SESN1, SESN2, p21, and GAPDH probes and the percentage of tumors with decreased expression of either SESN1 or SESN2 gene were determined.

To study the potential impact of the *SESN1* and *SESN2* genes in lung carcinogenesis, we analyzed mRNA expression of these genes using The Cancer Profiling Expression Array (Clontech) containing equal amounts of mRNA from matched human lung cancers and normal tissues from the same patient. The arrays were hybridized with ^32^P-labelled SESN1, SESN2, p21^Waf1/Cip1^, and GAPDH DNA probes. The expression levels of SESN1, SESN2, and p21^Waf1/Cip1^ mRNAs were diminished in the majority of human tumors (Figure 1). In contrast, the expression levels of GAPDH were either not changed or were increased in tumors. Therefore, we proposed that *SESN1* and *SESN2* are potential tumor suppressors of lung carcinogenesis as their expression appeared to be consistently downregulated in the majority of NSCLC tumors relative to control lung tissues.

### Inactivation of Sesn2 has a negative impact on lung tumor growth in the *Kras^G12D^*-based transgenic mouse model

**Figure 2:**
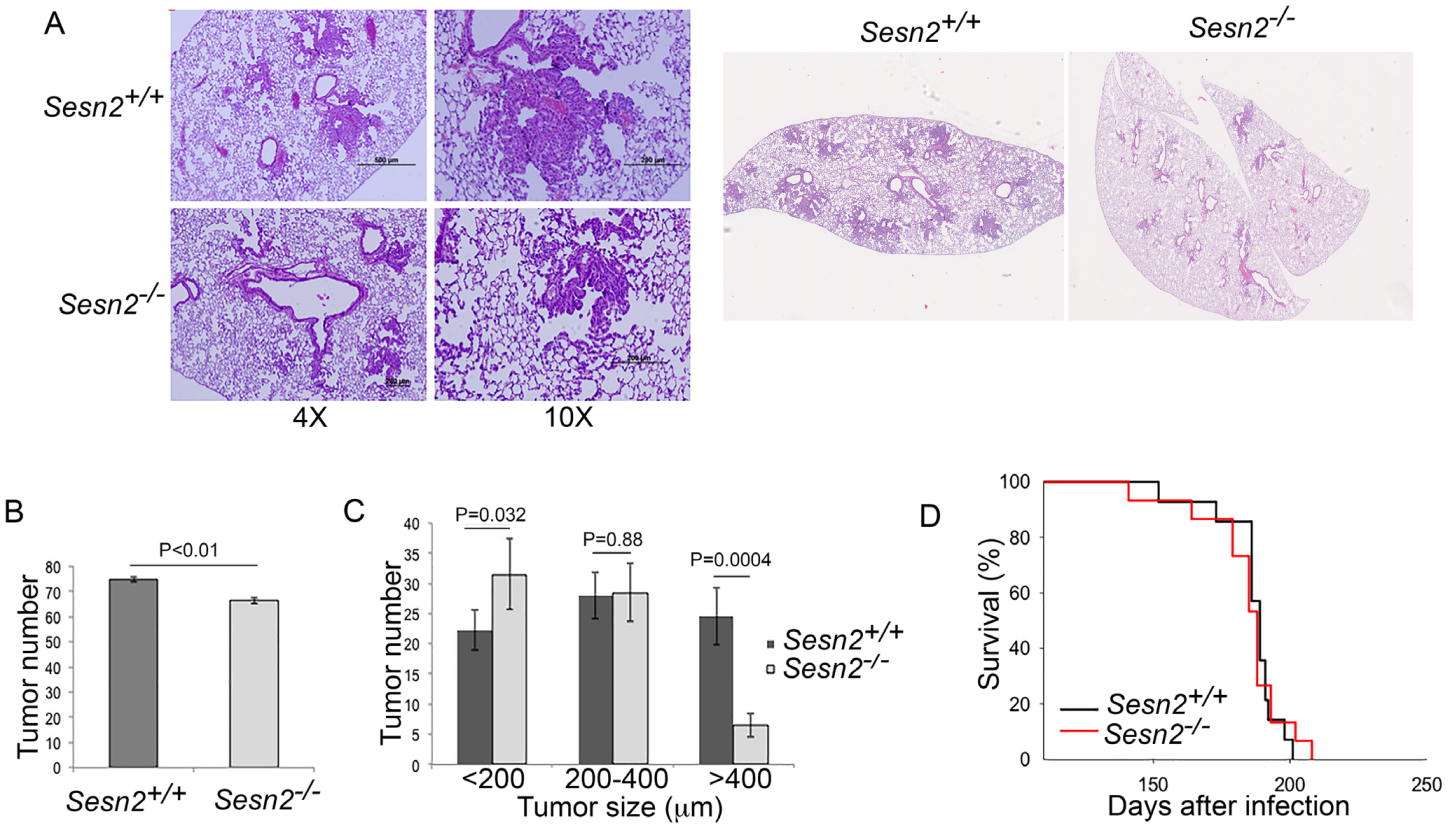
Sesn2 inactivation does not affect tumor initiation and life expectancy in tumor-bearing mice but slows down tumor growth. (**A**) Tumors from control and Sesn2-deficient mice. 2-month-old *Sesn2^+/+^;Kras^LSL-G12D^* and *Sesn2^-/-^;Kras^LSL-G12D^* mice were injected with Adeno-Cre intratracheally and analyzed 6 months later. The lung sections were stained with H&E. (**B**) The total number of tumors in *Sesn2^+/+^;Kras^LSL-G12D^* and *Sesn2^-/-^;Kras^LSL-G12D^* mice. (**C**) *Sesn2^-/-^* mice develop tumors of smaller size. In (A–C), 4 mice were analyzed per group. The data in (B) and (C) are presented as mean ± S.D. *P* values were calculated using two-tailed student’s *t*-test. (**D**) Tumor-bearing *Sesn2^+/+^* and *Sesn2^-/-^* mice have a similar lifespan. The mice (12 animals per each group) were injected with Adeno-Cre as in (A) and the lifespan was examined.

Based on the observations in Figure 1, we hypothesized that *SESN1* and *SESN2* may work as tumor suppressors and their inactivation may support lung carcinogenesis. To test the impact of SESTRINs on lung tumor growth, we generated *Sesn2^+/+^;Kras^LSL-G12D^* and *Sesn2^-/-^;Kras^LSL-G12D^* mice that develop lung tumors when injected intratracheally with recombinant adenovirus expressing Cre recombinase (Adeno-Cre) [4]. The sub-groups (4 mice in each group) of tumor-bearing mice were sacrificed 6 months after injection with Adeno-Cre to analyze the tumor size and number, while the other sub-groups (12 mice in each group) were followed to analyze the life span. Analysis of tumors by H&E staining demonstrated that the tumors from both mouse strains have similar morphology (Figure 2A), however, slightly fewer tumors were observed in the *Sesn2^-/-^;Kras^LSL-G12D^* mice (Figure 2B) and the tumors in the *Sesn2*-null mice were substantially smaller (Figure 2C). No notable difference in life expectancy was observed between *Sesn2^+/+^;Kras^LSL-G12D^* and *Sesn2^-/-^;Kras^LSL-G12D^* animals (Figure 2D). Therefore, Sesn2 has a positive impact on lung tumor growth during early steps of carcinogenesis but does not affect the life expectancy of tumor-bearing mice.

### Inactivation of Sesn1 does not have any additional effect on carcinogenesis in Sesn2-deficient mice

**Figure 3:**
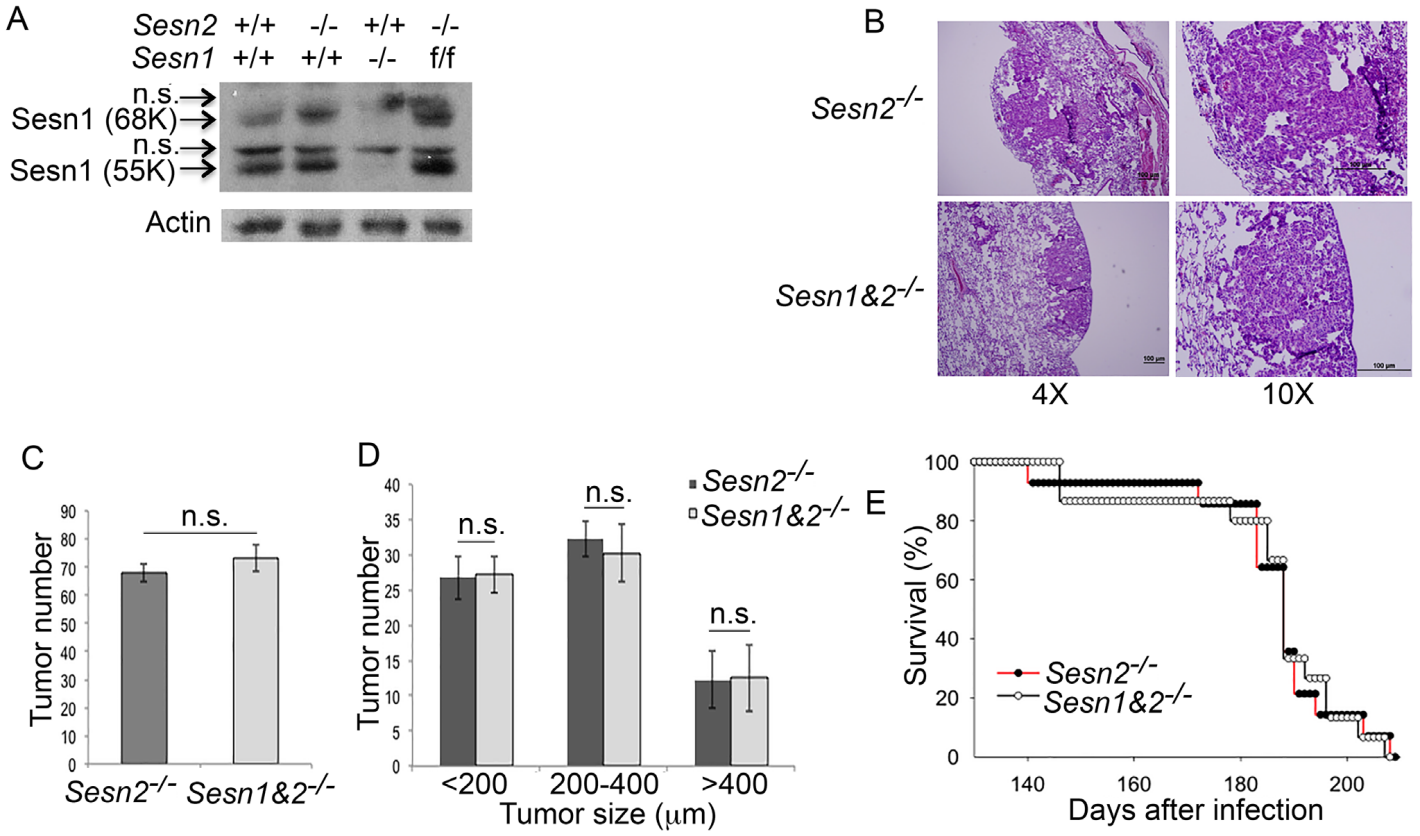
Inactivation of Sesn1 in *Sesn2^-/-^*;*Kras^LSL-G12D^* mice does not affect tumor phenotype. (**A**) Western blot analysis of lung lysates from control, *Sesn2^-/-^*, *Sesn1^-/-^*, and *Sesn1^F/F^* animals. (**B–D**) *Sesn1^-/-^; Sesn2^-/-^;Kras^LSL-G12D^* and *Sesn2^-/-^;Kras^LSL-G12D^* animals produce tumors of similar morphology (B), number (C), and size (D). 4 mice were analyzed per group. The data in (C) and (D) are presented as mean ± S.D. *P* values were calculated using a two-tailed student’s *t*-test; n.s. – not significant. (**E**) *Sesn2^-/-^;Kras^LSL-G12D^* and *Sesn1^-/-^;Sesn2^-/-^;Kras^LSL-G12D^* animals have a similar lifespan. 12 mice were analyzed per group.

In contrast to our prediction, *Sesn2* supports tumor growth and does not have any effect on life expectancy in the Kras-based cancer model. Therefore, we hypothesized that *Sesn1*, the other p53-inducible SESTRIN family member, may be responsible for tumor-suppressive effects of p53 in lung compensating for the effects of Sesn2 loss. To address the potential role of Sesn1 in lung carcinogenesis, we generated *Sesn1^-/-^* mice from a randomly targeted ES cell library (EUCOMM) where expression of the *Sesn1* gene is disrupted by the integration of a β-gal-neo cassette into the 2^d^ intron of the gene as described previously [30]. The absence of expression of Sesn1 protein (55K and 68K forms) was confirmed by immunoblotting (IB) (Figure 3A). The expression of Sesn1 can be restored by crossing mice with the mouse strain expressing the recombinase Flpase under control of the actin promoter, allowing us to use these mice for the following studies based on tissue-specific inactivation of the *Sesn1* gene (Figure 3A). *Sesn1^-/-^* mice were crossed with *Sesn2^-/-^;Kras^LSL-G12D^* mouse strain to generate double knockout *Sesn1^-/-^;Sesn2^-/-^;Kras^LSL-G12D^* mice. The cohorts of *Sesn2^-/-^;Kras^LSL-G12D^* and *Sesn1^-/-^;Sesn2^-/-^;Kras^LSL-G12D^* mice (16 mice per group) were injected with Adeno-Cre and size and the number of tumors (4 mice per group) as well as survival rates of the mice (12 mice per group) were analyzed as previously described. No significant differences in tumor morphology, size, and number, as well as life expectancy, were observed between *Sesn2^-/-^;Kras^LSL-G12D^* and *Sesn1^-/-^;Sesn2^-/-^;Kras^LSL-G12D^* mice, indicating that Sesn1 does not contribute to the suppression of tumor growth in Sesn2-deficient animals in the Kras^G12D^-based mouse model (Figure 3B–3D).

### Inactivation of Sesn2 inhibits AKT in mouse lungs but not in human lung adenocarcinoma cells

**Figure 4:**
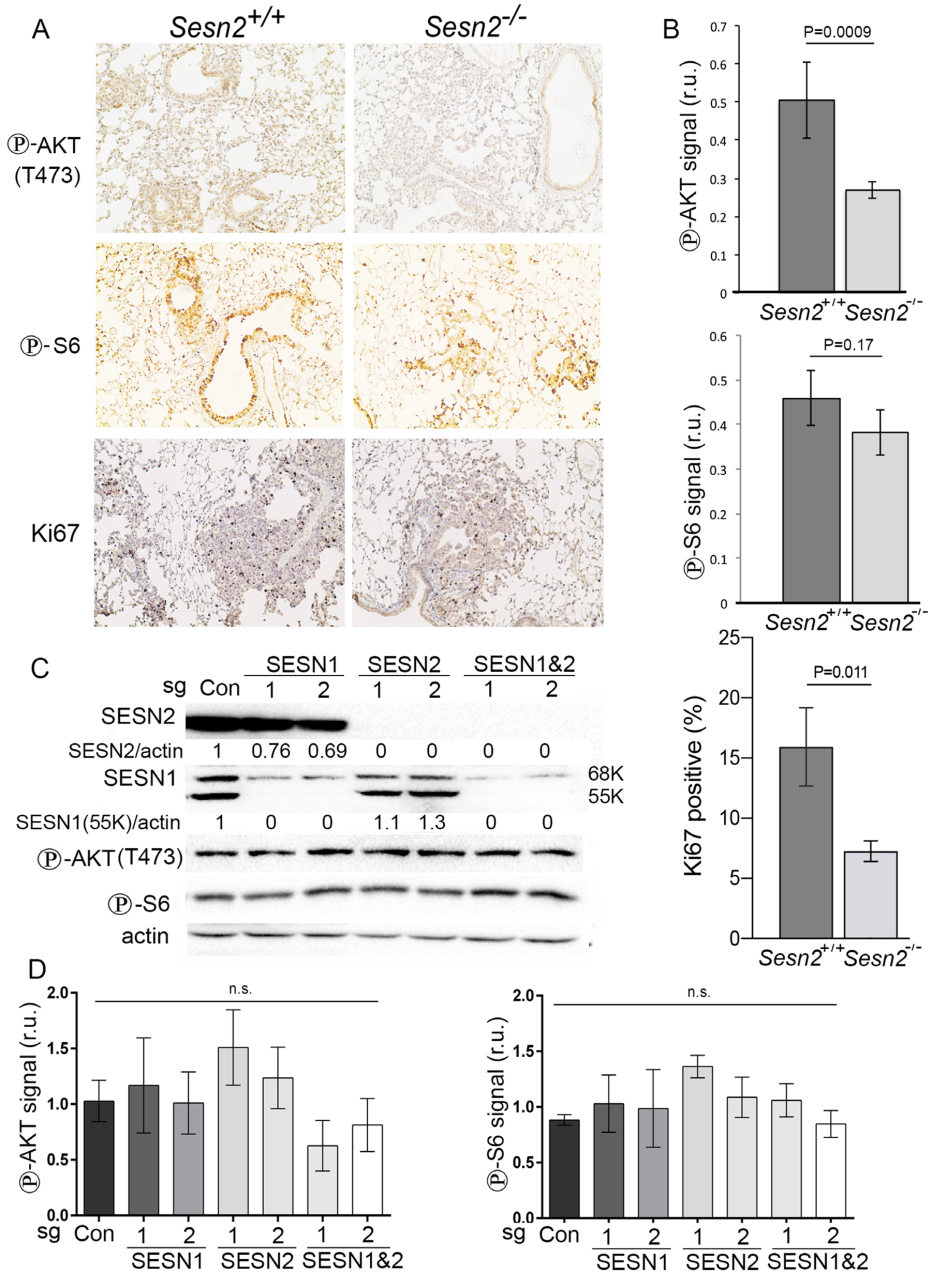
Inactivation of Sesn2 diminishes phosphorylation of AKT. (**A**) The sections of lung tissue from *Sesn2^+/+^;Kras^LSL-G12D^* and *Sesn2^-/-^;Kras^LSL-G12D^* mice were analyzed by immunohistochemistry with antibodies against phospho-AKT and phospho-S6 proteins. (**B**) The quantification of the intensity of the staining by the program ImageJ presented in relative units (r.u.). The data represent the mean ± S.D. *P* values were calculated using a two-tailed student’s *t*-test. (**C**) Western blot analysis of expression of SESN1 and SESN2 and phosphorylation of AKT and S6 in control A549 cells and cells where SESN1 and SESN2 were inactivated by 2 different CRISPR/Cas9 constructs. (**D**) Quantification of the relative intensity of phospho-AKT (Thr374) and phospho-S6 (Ser235/236). *P* values were calculated using one-way ANOVA followed by Tukey’s test for multiple comparisons (*n* = 3); n.s. – not significant.

To better understand the link between the inactivation of Sesn2 and suppression of tumor growth, we hypothesized that the positive impact of Sesn2 on tumor growth could be mediated by the activation of AKT kinase as the deficiency of Sesn2 might downregulate AKT activity required for tumor growth. To understand the role of Sesn2 in the regulation of AKT, we analyzed the phosphorylation of AKT by immunohistochemistry (IHC). We observed a substantial decrease in the levels of AKT phosphorylation on Ser473 in the lungs of *Sesn2^-/-^* mice as compared to *Sesn2^+/+^* controls (Figure 4A–4B). To define whether the activity of mTORC1 is also affected in the lungs of Sesn2-null mice, we also stained mouse lung sections with the antibodies against phospho-S6 (Ser235/236), the marker of mTORC1 activity. Unlike the results obtained with phospho-AKT staining, we did not observe any significant difference in the levels of phospho-S6 between control and Sesn2-deficient mice, indicating that the effects of Sesn2 on AKT phosphorylation are not mediated by the feedback loop through inhibition of mTORC1 kinase (Figure 4A–4B).

AKT can potently stimulate cell growth and proliferation [18]. Therefore, to confirm the potential impact of Sesn2 inactivation on proliferation of cells in lung tumors, we stained lung sections with an antibody against Ki67, the marker of cell proliferation. As shown in Figure 4A–4B, inactivation of Sesn2 decreases the number of proliferating cells that potentially contributes to the retardation of tumor growth in the Sesn2-null mice.

To study whether inactivation of SESN1/2 affects phosphorylation of AKT in human lung cancers, A549 cell lines inactivated for *SESN1*, *SESN2*, or both genes using CRISPR/Cas9 constructs were prepared and immunoblots confirmed that the respective genes were inactivated (Figure 4C). Surprisingly, we did not observe any significant changes in the levels of AKT and S6 phosphorylation in the SESN1/2-deficient cells, which indicates that the AKT-mTORC1 pathway in advanced lung tumor cells might not be dependent on the *SESN1/2* genes (Figure 4C–4D).

### Inactivation of SESN1 and SESN2 in human lung adenocarcinoma A549 cells supports cell proliferation and provides resistance to glucose starvation

**Figure 5:**
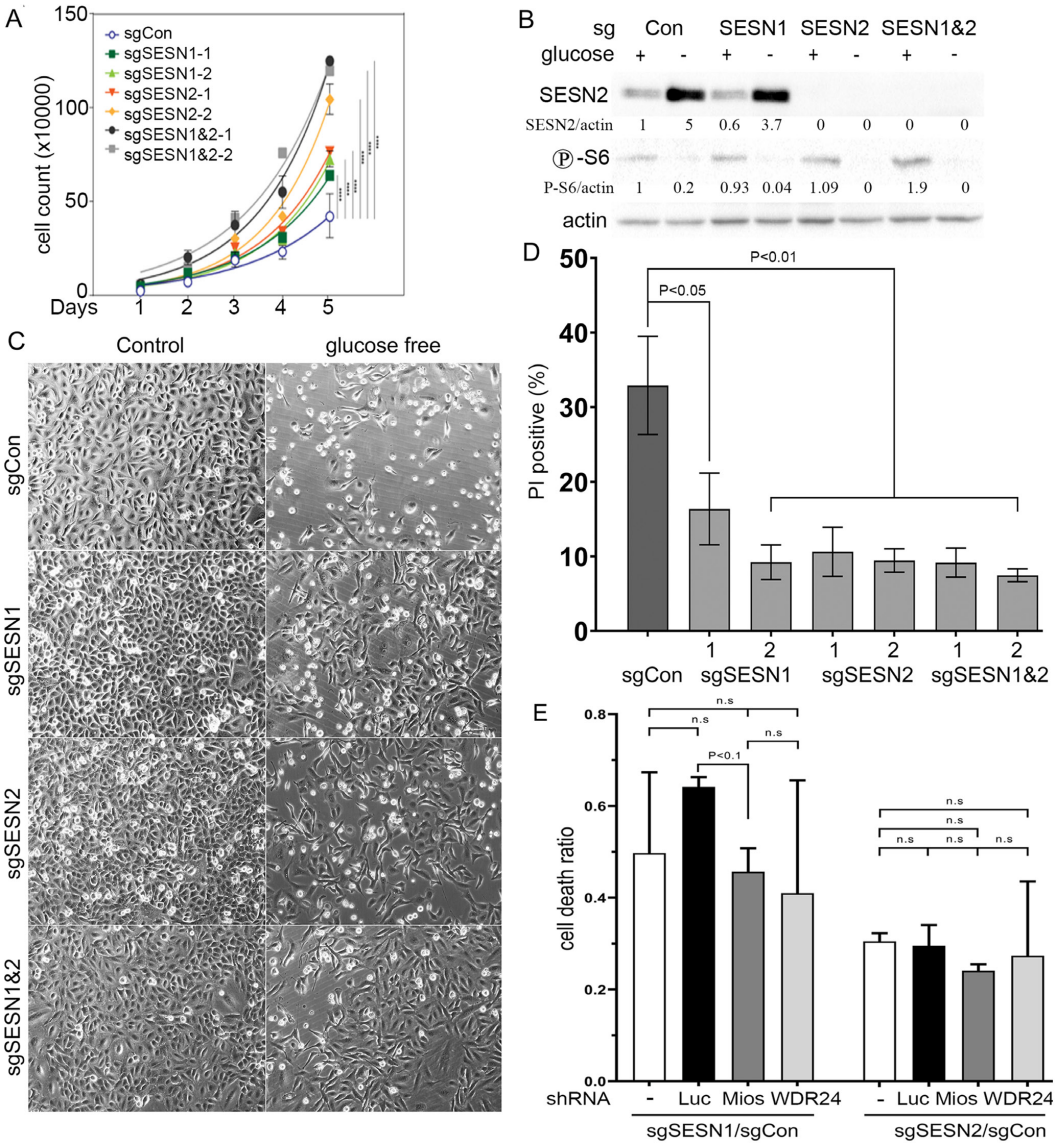
Inactivation of SESN1 and/or SESN2 in lung adenocarcinoma A549 cells stimulates cell proliferation rate and increases resistance to glucose starvation. (**A**) Cells of indicated genotypes were plated onto 6-well plates (50,000 cells/well) and counted every 24 h for 5 days. (**B**) Western blot analysis of SESN2 expression and S6 phosphorylation of cells of the indicated genotypes incubated 18 h in glucose-free medium. (**C**) A549 cells with inactivation of SESN1 and/or SESN2 are resistant to cell death in glucose-free medium. Cell death was analyzed by phase-contrast microscopy. (**D**) Analysis of PI-positive cells incubated 24 h in glucose-free medium by flow cytometry. Data are presented as mean ± SEM. *P* values were calculated relative to control using one-way ANOVA followed by correction for multiple comparisons using Dunnett correction (α = 0.05) in three independent experiments. (**E**) SESN1 and SESN2 support cell death in a GATOR2-independent manner. Mios and WDR24 were silenced in control, SESN1- and SESN2- deficient cells and the levels of cell death were evaluated by flow cytometry analysis of PI inclusion. The data are presented as a ratio of the cell death in sgSESN1/sgControl(Con) or sgSESN2/sgCon cells in the presence of control shRNA (shLuc), shMios, or shWDR24. In the experiments (B–E) cells were incubated with glucose-free DMEM supplemented with L-glutamine, penicillin/streptomycin, and 10% dialyzed fetal bovine serum.

We have previously reported that silencing of SESN1 or SESN2 stimulates the growth of lung adenocarcinoma A549 tumor xenografts in nude mice [7], which is in contrast with the negative effect of SESTRINs’ inactivation on tumor growth in the *Kras^LSL-G12D^*-based mouse model. Presumably, inactivation of SESN1 or SESN2 can benefit advanced tumors supporting cell proliferation, protecting against cell death, or contributing to other aspects of carcinogenesis. We analyzed whether inactivation of the *SESN1* and/or *SESN2* genes may affect cell proliferation that can be responsible for the acceleration of tumor growth. We observed that cells with inactivation of either SESN1 or SESN2 have an accelerated proliferation rate as compared to control. Moreover, the cells with the double knockout of SESN1 and SESN2 showed the fastest proliferation rate among all tested cell lines (Figure 5A).

Tumor growth is often associated with a limited supply of nutrients such as glucose and amino acids and SESTRINs may be involved in the regulation of cell death under such conditions [12, 24]. The incubation of A549 cells in glucose-free medium leads to the activation of SESN2 expression (Figure 5B). To study the potential link of the activation of SESTRINs with the activity of mTORC1 kinase, we analyzed mTORC1-regulated S6 phosphorylation and observed suppression of mTORC1 activity in all tested cell lines regardless of SESN1 and SESN2 expression (Figure 5B). We analyzed the levels of cell death in the control, SESN1-, SESN2- and double SESN1&2- deficient cells by microscopy and flow cytometry and observed that inactivation of the *SESN1* and/or *SESN2* genes protect A549 cells against cell death activated by glucose starvation as demonstrated by PI staining (Figure 5C–5D). Analysis of cell death at the early stages of cell death after 20 h of incubation in glucose-free medium by Annexin V; PI staining demonstrated that most of the dying cells were PI-positive and the number of apoptotic Annexin V-positive; PI-negative cells were extremely low, indicating that cells die through a mechanism different from apoptosis (Supplementary Figure 1). We also analyzed cell cycle distribution in control and SESN1/2-null cells incubated in normal and glucose-free medium and found that a higher number of SESN1/2-null cells were observed in the G0/G1 phase of the cell cycle and less in the S-phase of the cell cycle in glucose-free medium as compared to control cells, potentially contributing to the effects of SESTRINs on cell death (Supplementary Figure 2).

To define whether the effects of SESTRINs on cell death induced by glucose starvation may be mediated by the GATOR2 protein complex, the key interactor of SESTRINs [20, 31], we silenced the major components of GATOR2 – Mios and WDR24 in control, SESN1- or SESN2- deficient cells and analyzed the ratio in the levels of cell death between either SESN1- or SESN2-deficient and control cells expressing no shRNA, control shLuc, shMios, or shWDR24. We observed the similar sgSESN2/sgControl(Con) ratio in cell death in the control cells and the cells with knockdown of GATOR2 proteins and even slightly decreased sgSESN1/sgCon cell death ratio between shLuc and GATOR2-silenced cells (Figure 5E).

## DISCUSSION

The genes encoding SESN1 and SESN2 proteins are potential candidates for tumor suppressors in the lung. This assumption is supported by several important observations published by our and other groups:

I) Both genes are bona fide transcriptional targets of tumor suppressor p53 that is inactivated in most human cancers [32]; II) Silencing of either SESN1 or SESN2 in cancer cells causes DNA oxidation, mutagenesis, and genomic instability [7, 33], the major contributors to carcinogenesis [34]; III) SESTRINs are inhibitors of mTORC1 kinase that plays an important role in regulation of cell growth, proliferation, energetic metabolism, and angiogenesis and the mTORC1-regulated pathway is activated in the majority of cancers [17]; IV) SESN2 expression in NSCLC is a favorable biomarker in patients [35]; V) SESN2 supports cell death in lung cancer cells in response to the activation of death receptors by cytokines that can be used by the systems of immune surveillance [29]; and VI) Silencing of either SESN1 or SESN2 expression accelerates growth of A549 tumors in immunocompromised nude mice [7]. However, the precise role of SESN1/2 in the regulation of lung carcinogenesis needs to be verified in more detailed studies based on lung cancer animal models.

Our current data indicate that the expression levels of the *SESN1* and/or *SESN2* genes are often diminished in human lung cancers and suppression of SESN1/2 may support tumor growth and/or progression through such mechanisms as stimulation of cell growth, proliferation, mutagenesis, and angiogenesis [25]. Downregulation of the expression of the *SESN1/2* genes can be caused by several factors often associated with lung carcinogenesis. The main mechanism involves inactivation of p53, the master regulator of *SESN1* and *SESN2* [7, 11, 36], which is mutated in the majority of lung and other types of cancers [32]. Two other processes contributing to carcinogenesis lead to suppression of SESTRINs’ expression. As we showed recently, a mutant form of histone methyltransferase EZH2, responsible for transcriptional repression of multiple genes in cancer cells, inhibits expression of SESN1 in lymphomas and restoration of SESN1 expression in the tumors leads to suppression of lymphomagenesis in mice [37]. EZH2 is overexpressed in many human lung cancers, correlating with poor prognosis [38], and EZH2 may be responsible for the downregulation of SESN1 expression in lung cancers. Interestingly, the KRAS proto-oncogene, often activated by mutation in lung tumors, stimulates the expression of EZH2 [39] that can also lead to SESN1 downregulation. Accordingly, the overexpression of mutant forms of RAS gene family members strongly suppresses expression of the *SESN1* gene [6, 33]. EZH2 silencing in lung adenocarcinoma cells attenuates cell cycle progression, cell growth and invasion [40, 41] that can be linked to the re-activation of SESN1. A novel mechanism of suppression of SESN2 was demonstrated in neuroblastoma cells and is mediated by lysine-specific demethylase LSD1 [42]. High expression of LSD1 is associated with poor prognoses for lung cancer patients and LSD1 inhibition by small molecules or siRNA leads to suppression of cell growth, invasion, and migration [43].

In contrast to our previous studies based on A549 tumor xenografts [7], the inactivation of Sesn2 slows down tumor growth in the model of *Kras^LSL-G12D^*-driven carcinogenesis in mice, however, has no effects on life span (Figure 2B–2D). Inactivation of *Sesn1* has no additional effects on tumor growth and the lifespan of *Sesn2^-/-^;Kras^LSL-G12D^* mice (Figure 3C–3E). These data indicate that SESTRINs probably do not play an important role in the suppression of early stages of lung carcinogenesis and might even support tumor growth during these stages. However, it is unlikely that SESTRINs play a strong positive role during early steps of cancer development as the activation of these genes might be also associated with many processes adverse to the tumors such as inhibition of mTORC1 or activation of cell death.

Nevertheless, certain levels of SESN2 expression during early steps of carcinogenesis might be required to support the activity of AKT kinase, the critical positive regulator of glucose transport, anabolism, and cell viability that contributes to growth and proliferation of tumor cells [18]. SESTRINs regulate AKT through at least three distinct mechanisms including the inhibition of the negative feedback loop stimulated by hyperactive mTORC1, the control of PTEN localization on the cytoplasmic membrane, and direct activation of mTORC2 via protein-protein interactions [25]; therefore, this pathway might be very important for the regulation of cell homeostasis by SESTRINs. Analysis of tumors by immunohistochemistry indicates that *Sesn2*-deficient tumors have lower levels of AKT activity than *Sesn2*-proficient counterparts (Figure 4A–4B). Interestingly, similar effects, showing that *Sesn2* inactivation leads to a decrease of AKT phosphorylation, were observed in our previous studies on mouse embryonic fibroblasts [26]. However, phosphorylation of AKT and S6 was not changed in the lung adenocarcinoma A549 cells with the knockout of the SESN1 and/or SESN2 genes (Figure 4C–4D), indicating that pro-oncogenic signaling pathways activated during carcinogenesis may overcome the need for SESTRINs to support the high activity of AKT. SESN1 and SESN2 also do not play a substantial role in the control of mTORC1, as indicated by similar phosphorylation levels of ribosomal S6 protein in control and SESN1/2-deficient cells. Thus, SESTRINs might regulate AKT phosphorylation in non-transformed cells or in cells at the early stages of carcinogenesis but not at the advanced stages. Therefore, inactivation of SESN1/2 at the late stages of lung carcinogenesis might not contribute to suppression of AKT activity and may benefit cancer cells through mechanisms yet to be defined. Particularly, inactivation of SESN1/2 could contribute to the late steps of carcinogenesis via support of mutagenesis, genomic instability, or resistance to cell death [13]. The model of carcinogenesis based on the activation of only the *Kras^LSL-G12D^* oncogene, used in our study, precludes examining the late stages of carcinogenesis as most of the mice die from asphyxia and the tumors rarely progress to the late metastatic stage [4]. Therefore, the impact of SESTRINs on cancer progression needs to be re-examined in more appropriate mouse models that develop tumors capable of progressing to the late stages, yet still preserving some activities of p53.

We have previously shown that SESN2 suppresses cell proliferation and stimulates cell death in tumor cells in response to DNA-damage and cytokines, however, it can also support cell viability under particular stress conditions in certain cell types [11, 29]. Accordingly, we demonstrated that the inactivation of the SESN1 and/or SESN2 genes by the CRISPR/Cas9 system accelerates cell proliferation in A549 cells (Figure 5A) and this mechanism may be responsible for tumor growth. The growth of tumors is also associated with the deprivation of nutrients such as glucose and amino acids. Glucose starvation not only prevents sufficient energy production but also induces endoplasmic reticulum (ER) stress followed by the unfolded protein response [12, 44]. Prolonged ER stress induces cell death via the activation of the transcription factor CHOP or other not well-characterized mechanisms [44]. We have found that in A549 cells SESN1 and SESN2 contribute to the induction of cell death in glucose-starved cells. These effects are not caused by the inhibition of mTORC1 as glucose starvation suppresses mTORC1 activity in a SESN1/2-independent manner (Figure 5B–5D). Also, our experiments demonstrate that SESN1/2-dependent activation of cell death is not dependent on the GATOR2 complex, a well-established SESTRINs’ interactor (Figure 5E) [20, 31].

It was previously described that glucose deprivation may stimulate cell death through caspase-dependent and caspase-independent mechanisms [16, 45]. Our studies based on Annexin V; PI staining show that even after 20 h incubation with glucose-free medium, when we start detecting the earliest events of cell death, most cells detected by flow cytometry analysis were PI-positive (Supplementary Figure 1) and only a negligible number of apoptotic Annexin V-positive, PI-negative cells were detected. We also were not able to detect the cleaved form of PARP, the hallmark of apoptotic cell death (data not shown). Therefore, we concluded that the type of cell death modulated by SESN1/2 is not caspase-dependent apoptosis but some other form of cell death. This type of cell death is probably mediated by induction of the ER stress response and might be linked with the hexamine biosynthesis pathway, as was previously reported for the cells expressing oncogenic KRAS [16, 46]. We also found that inactivation of SESN1/2 proteins leads to the accumulation of cells in the G0/G1 phase and reduction in cell number in the S phase of the cell cycle that might contribute to protection against cell death (Supplementary Figure 2).

The important role of SESN1/2 in the facilitation of cell death in A549 cells is in contrast with our previous data, obtained on immortalized mouse embryonic fibroblasts and some tumor cell lines. It was demonstrated previously that SESN2 plays a protective role against glucose deprivation, supporting respiration and ATP production under stress conditions [16]. The positive effect of SESN1 and SESN2 on the activation of cell death in A549 cells can also be explained by the direct contribution of SESTRINs to the activation of cell death machinery potentially involving extensive mitophagy that can lead to failure to support pro-survival functions causing necrotic cell death [47–51]. Therefore, a detailed analysis of whether SESTRINs contribute to cell death through the regulation of metabolic pathways, cell proliferation, or direct activation of cell death in lung adenocarcinoma cells will help us to better understand the role of SESTRINs in carcinogenesis in future studies.

Therefore, SESTRINs may suppress proliferation and support cell death in cancer cells in response to nutrient deficiency and cytokines and the signals associated with SESN1/2-dependent cell death may also stimulate an immune response aiming to eradicate tumor cells. Inactivation of SESN1/2 may give cancer cells additional advantages to survive in the harsh conditions of tumor-host interactions. Considering SESTRINs as potential drug targets it would be important to understand which factors determine whether SESTRINs play an anti-survival or a pro-survival role in cancer cells, which will allow us to choose the best strategy to treat a particular type of cancer based on the levels of SESTRINs’ expression. SESTRINs may also be determinants of sensitivity to anti-cancer treatments as they are activated in response to radiotherapy and many chemotherapeutic drugs and may support cell death in response to certain treatment conditions.

## MATERIALS AND METHODS

### Analysis of gene expression

The Cancer Profiling Expression Array (Clontech) was hybridized with ^32^P-CTP labeled cDNA probes for SESN1, SESN2, p21^Waf1^, and GAPDH as described [11].

### Mice

All mice were on C57/BL background. *Sesn2^-/-^* mice were described previously [15]. *Sesn1^-/-^* mice were generated as described [30]. *Kras^LSL-G12D^* mice were characterized previously [4]. To induce lung carcinogenesis 2-month-old mice (16 animals per group) were injected with Adeno-Cre intratracheally with the titer of 2.5 × 10^7^ PFU/mouse and tumors were analyzed 6 months later [4]. All animal studies were overseen by Virginia Commonwealth University Institutional Animal Care and Use Committee (IACUC).

### Cell culture and treatment

Cells were cultured in DMEM containing 4.5 g/l glucose, supplemented with L-glutamine (2 mM), penicillin/streptomycin, and 10% fetal bovine serum. For glucose starvation cells were incubated in glucose-free, sodium pyruvate-free DMEM (Gibco) supplemented with L-glutamine (2 mM), penicillin/streptomycin, and dialyzed 10% fetal bovine serum.

### Constructs, cells, and lentiviral infection

The targeted sequences for sgSESN2-1 and sgSESN2-2 were 5′-CTCGGAGTCCGCCACGATCA-3′ and 5′-AGAGCCTCGAGCAGCACCTG-3′, respectively [16, 52]. The targeted sequences for sgSESN1-1 and sgSESN1-2 were 5′-CTACATTATCGTCACTACAT-3′ and 5′-AATGTAGTGACGATAATGTA-3′, respectively. The targeted sequences of shLuc were described previously [15]. The targeted sequence of shMios: 5′-TCTCAATGTGGTAGCAATGGC-3′ and shWDR24: 5′-CCAATGAGGATAACGAGGAAA-3′. A549 were obtained from American Type Culture Collection (ATCC). The cells were cultured in DMEM supplemented with 10% FBS, 2 mM L-glutamine, and penicillin/streptomycin. To generate SESN1/2-deficient A549 cells, the cells were infected with indicated lentiviral constructs and selected in the medium with puromycin (1 μg/ml) as described previously [16]. The single cells were plated on a 96-well plate using cell sorter and were allowed to form colonies 3–4 weeks after plating. The expression of SESN1 or SESN2 genes in the colonies was analyzed by immunoblot (IB) and 15 SESN1- or SESN2- negative clones were pulled together to form SESN1/2-negative multi-cloned cell cultures to be used in the following studies to avoid clone-specific effects. To generate SESN1&2 double knockouts, the SESN1-deficient cells were infected with recombinant lentivirus expressing sgSESN2-1 and the cell culture was generated as previously described. To generate cells with knockdown of Mios or WDR24, cells were infected with corresponding recombinant lentiviral vector and 48 h later selected with hygromycin (350 μg/ml) for 2 weeks.

### Cell lysis and immunoblot analysis

For IB analysis of tissues mice were euthanized, lungs were placed in RIPA-SDS buffer, containing protease and phosphatase inhibitors (Roche), and homogenized on ice. For lysis of cells in culture, cells were placed on ice, washed twice with ice-cold PBS, and lysed in RIPA-SDS buffer containing protease and phosphatase inhibitors (Roche). Protein concentration was estimated with Pierce BCA Protein Assay Kit (Thermo scientific) and 30 μg of protein was used for each load. Rabbit anti-phospho (Ser473) AKT and anti-phospho (Ser235/236) antibodies were from Cell Signaling Technology. Mouse anti-β-actin antibody was from Santa Cruz Inc. Rabbit anti-SESN2 antibody was from Proteintech Inc. Rabbit anti-SESN1 antibody was previously described [26, 37]. The intensities of bands were calculated by ImageJ.

### Immunohistochemistry (IHC)

The mice were euthanized, their lungs were removed, fixed, and paraffin-embedded and sections were analyzed as described [53]. For the analysis of tumor morphology, the lung sections were stained with H&E. For the analysis of AKT and S6 phosphorylation and Ki67 expression, the slices were stained with corresponding antibodies followed by peroxidase staining. The intensities of phospho-AKT and phospho-S6 signals were analyzed by ImageJ. For the analysis of proliferation in lung tumor sections, the percentages of Ki67-positive cells per total number of cells in tumors were calculated.

### Statistical analysis

Statistical analysis was performed with GraphPad Prism software version 5.0 (GraphPad Software, La Jolla, CA) using two-tailed Student’s *t*-test or one-way ANOVA followed by Tukey’s test for multiple comparisons. Statistical significance was defined as *P* < 0.05. Results are presented as mean ± standard deviations or standard errors as indicated in the legends.

## SUPPLEMENTARY MATERIALS



## Abbreviations

mTORC1/2: mammalian target of rapamycin complex 1/2
ROS: reactive oxygen species
NSCLC: non-small cell lung cancer
