# Supplementary material for: p53-inducible SESTRINs might play opposite roles in the regulation of early and late stages of lung carcinogenesis
Source: Oncotarget. 2019 Dec 10;10(65):6997–7009. doi: 10.18632/oncotarget.27367 (PMC6916756; doi:10.18632/oncotarget.27367)
Supplement: Supplementary file 1 [file oncotarget-10-6997-s001.pdf]

p53-inducible SESTRINs might play opposite roles in the regulation of early and late stages of lung carcinogenesis

SUPPLEMENTARY MATERIALS

MATERIALS AND METHODS

Cell cycle analysis

A day before treatment 100,000 cells per well were plated onto 12-well plates. The cells were washed with PBS before treatment and 1 mL of glucose-free medium replaced the culture medium. Cells were incubated in humidified 37°C, 5% CO2 incubator. After 24 hours of incubation the glucose-free medium was extracted into 1.5 mL centrifuge tubes. Immediately after cells were washed with 200 µL PBS and the PBS was transferred to the corresponding 1.5 mL tubes. After the wash 200 µL of trypsin-EDTA was placed into each well and the plates were left in the incubator for up to 10 minutes. Subsequently, the cells were resuspended in trypsin and transferred into corresponding 1.5 mL tubes. The tubes were centrifuged at 300 g at 4°C for 15 minutes. After centrifugation the supernatant was discarded and the cellular pellet was resuspended in cold PBS. After two washes and centrifugations with PBS the washed cellular pellet was resuspended in 400 µL PBS. 800 µL of ice-cold 100% ethanol was slowly added. Cells were left in this mix at 4°C for at least 2 hours. The ethanol-treated cells were then resuspended, centrifuged at 1000 × g for 5 minutes. and washed with PBS. Finally, the cellular pellet was resuspended in 200 µL PBS containing 50 µg/mL of PI and 50 µg/mL of RNAse and incubated in this solution at 37°C in the dark for 30 minutes. The samples were then analyzed using BD Accuri C9. Samples were thoroughly resuspended prior to analysis to avoid aggregates. BD Accuri C9 software was used to analyze the data.

Annexin V apoptosis analysis

Cells were harvested as previously described. After harvesting the cells were washed two times in the Annexin V-binding buffer (AVBB, 10 mM HEPES, 150 mM NaCl, 5 mM KCl, 1 mM $MgCl_2$, 1.8 mM $CaCl_2$, pH 7.4). The cells were then stained in 50 µL AVBB containing 2 µg/mL Annexin V-FITC, left to stain at room temperature in the dark for 15 minutes, and were centrifuged and washed twice in AVBB. Finally, each sample was resuspended in 500 µL AVBB. Immediately before being analyzed with BD Accuri C9, the cells were counterstained with propidium iodide (100 µg/mL). BD Accuri C9 software was used to analyze the data.

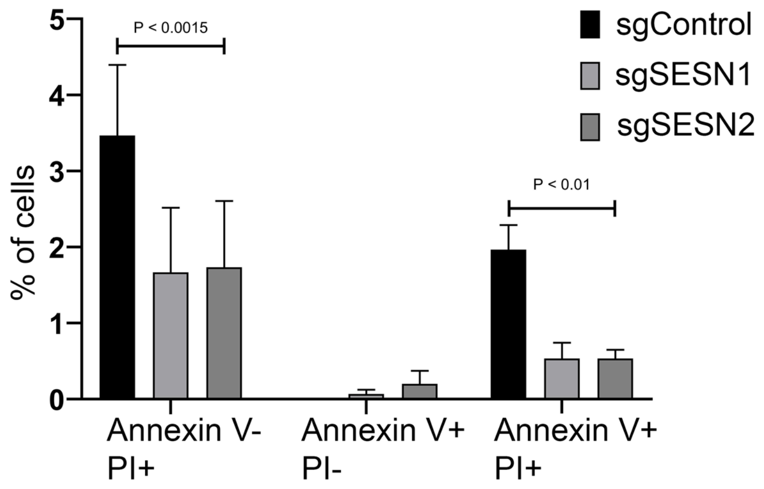

Supplementary Figure 1: Inactivation of SESN1 or SESN2 protects against necrotic but not apoptotic cell death induced by glucose starvation. Control, SESN1- and SESN2- deficient cells were incubated in glucose-free medium for 20 h, stained with AnnexinV;PI and analyzed by flow cytometry. Statistical analysis was conducted using two-way ANOVA with Tukey's multiple comparisons test ($\alpha = 0.05$, GraphPad Prism 8). Data represent the mean percentage of the corresponding cells in the population ±SD ($n = 3$).

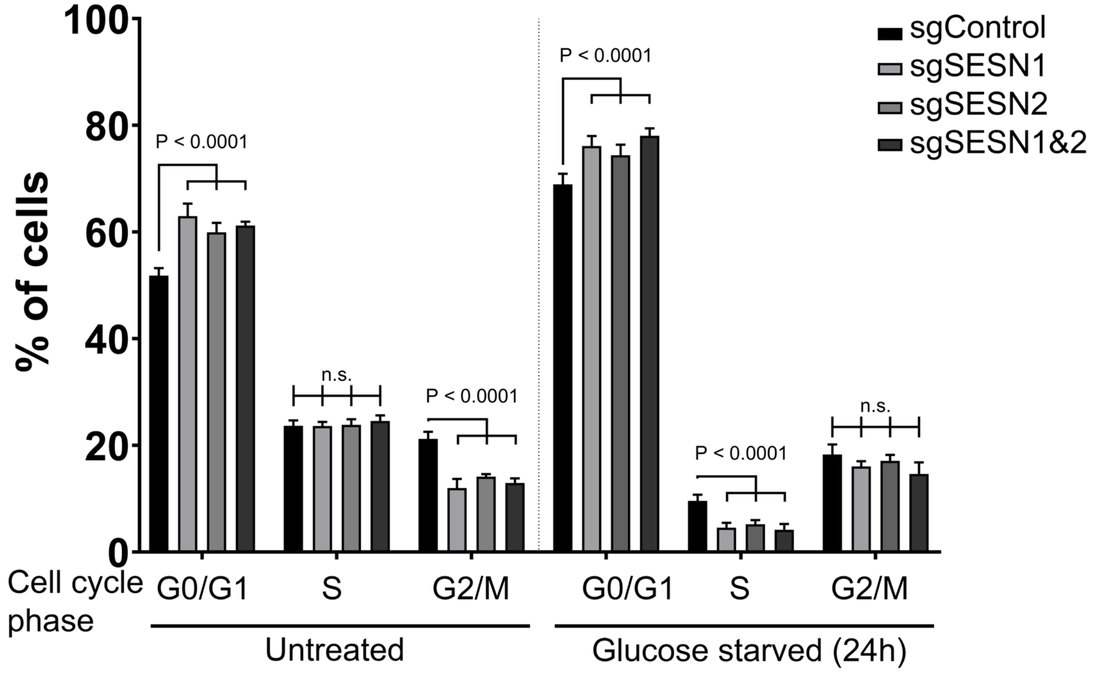

Supplementary Figure 2: Cell cycle distribution of control cells and SESN1-, SESN2- and SESN1&2-null cells in control conditions and after glucose deprivation. Cells were incubated in glucose-free medium for 24 hours, fixed with 70% ice-cold EtOH, and stained with PI. Statistical analysis was conducted using two-way ANOVA with Tukey's multiple comparisons test ($\alpha = 0.05$, GraphPad Prism 8). Data represent the mean percentage of the corresponding cells in the population ±SD ($n = 6$).
